# miR-409 and miR-411 Modulation in the Adult Brain of a Rat Model of Depression and After Fluoxetine Treatment

**DOI:** 10.3389/fnbeh.2020.00136

**Published:** 2020-08-07

**Authors:** Patrícia Patrício, António Mateus-Pinheiro, Nuno Dinis Alves, Mónica Morais, Ana João Rodrigues, João Miguel Bessa, Nuno Sousa, Luísa Pinto

**Affiliations:** ^1^Life and Health Sciences Research Institute (ICVS), School of Medicine, University of Minho, Braga, Portugal; ^2^ICVS/3B’s—PT Government Associate Laboratory (AL), Braga/Guimarães, Portugal

**Keywords:** chronic stress, depression, fluoxetine, miR-409, miR-411, neural plasticity

## Abstract

Depression is a chronic debilitating disorder predicted to affect around 20% of the world population. Both brain and peripheral changes, including neuroplastic changes have been shown to occur in the brains of depressed individuals and animal models of depression. Over the past few decades, growing evidence has supported the role of miRNAs as regulators of critical aspects of brain plasticity and function, namely in the context of depression. These molecules are not only highly expressed in the brain, but are also relatively stable in bodily fluids, including blood. Previous microarray analysis from our group has disclosed molecular players in the hippocampal dentate gyrus (DG), in the context of depression and antidepressant treatment. Two miRNAs in particular—miR-409-5p and miR-411-5p—were significantly up-regulated in the DG of an unpredictable chronic mild stress (CMS) rat model of depression and reversed by antidepressant treatment. Here, we further analyzed the levels of these miRNAs along the DG longitudinal axis and in other brain regions involved in the pathophysiology of depression, as well as in peripheral blood of CMS-exposed rats and after fluoxetine treatment. The effects of CMS and fluoxetine treatment on miR-409-5p and miR-411-5p levels varied across brain regions, and miR-411-5p was significantly decreased in the blood of fluoxetine-treated rats. Additional bioinformatic analyses revealed target genes and pathways of these miRNAs related to neurotransmitter signaling and neuroplasticity functions; an implication of the two miRNAs in the regulation of the cellular and molecular changes observed in these brain regions in depression is worth further examination.

## Introduction

Major Depression (MD) is a chronic debilitating disorder predicted to affect more than 300 million people worldwide. Moreover, the World Health Organization has rated it as the leading cause of disease burden in developed countries since 2017 (GBD 2017 Disease and Injury Incidence and Prevalence Collaborators, 2018). Despite the available pharmacological treatments, less than 70% of all patients that are currently treated with available antidepressants present full remission (Insel and Wang, 2009).

Depression most likely results from a complex interaction between genetic predisposition and environmental factors, such as early life experiences, life events, and chronic stress (Mandelli and Serretti, 2013). Though imbalances in the levels of monoaminergic neurotransmitters have long been assumed as central in the pathophysiology of depression, current knowledge puts forward many other systems as relevant for its pathophysiology and treatment. Both central and peripheral substrates, such as neuroimmune and neuroendocrine systems, neuroplasticity, and the gut microbiome are acknowledged substrates of the disease; however, it is still challenging to integrate the observed imbalances to interpret the full spectrum of behavioral outcomes observed in depressed individuals (Krishnan and Nestler, 2010; Schmidt et al., 2011; Galea et al., 2013).

Neuroplasticity changes, in particular, have been consistently described in the brain of depressed individuals and animal models of depression (Stockmeier et al., 2004; Sahay et al., 2011). These changes occur in several adult brain regions that play an important role in behavioral processes somehow related to the pathophysiology of the disease, from learning and memory to anxiety and mood. The hippocampus, one of the most widely studied brain regions in this context, is a glucocorticoid receptors-rich region extending along a Spatio-temporal axis, and particularly sensitive to the effects of chronic stress, a major precipitating factor for depression (Anacker et al., 2011; Egeland et al., 2015). Within this brain region lies the hippocampal dentate gyrus (DG), a functionally heterogeneous structure and one of the adult brain regions where new cells can be generated from resident progenitors (Kheirbek et al., 2013; Tanti and Belzung, 2013).

Previous microarray analysis from our group has disclosed a set of molecular players differently expressed in the hippocampal DG in the context of depression and antidepressant treatment (Patrício et al., 2015). Two miRNAs in particular—miR409 and miR411—were shown to be significantly up-regulated in the DG of an unpredictable chronic mild stress (CMS) rat model of depression, and reversed by different antidepressants treatment, namely fluoxetine, imipramine, tianeptine and agomelatine (Patrício et al., 2015). These two miRNAs, in particular, have not yet been reported in human studies of depressed patients.

miRNAs are a family of small (19–25 nucleotides) highly conserved non-coding RNAs, that regulate gene expression at the post-transcriptional level. miRNAs bind primarily to the 3’ UTR of mRNAs leading to mRNA destabilization or repressing translation. Over the past few years, growing evidence has supported the role of miRNAs as regulators of critical aspects of neuroplasticity and brain function (Dreyer, 2010; Im and Kenny, 2012; O’Carroll and Schaefer, 2013; Dubes et al., 2019). Additionally, alterations in miRNAs levels have been reported in several neuropsychiatric disorders, including depression and as targets for antidepressant treatment (Hansen and Obrietan, 2013; Issler et al., 2014; Gururajan et al., 2016; O’Connor et al., 2016). These molecules are not only highly expressed in the brain, but are also relatively stable in bodily fluids, including blood (Gheysarzadeh et al., 2018). The association between changes in miRNAs in bodily fluids, such as blood and cerebrospinal fluid (CSF), and brain tissue has generated great interest in the field, as these may represent potential biomarkers for disease (Li et al., 2013; Camkurt et al., 2015; Gururajan et al., 2016; Lopez et al., 2018).

Hence, the present work aimed to further analyze the levels of these two miRNAs along the septotemporal axis of the DG as well as in other brain regions involved in the pathophysiology of depression, including the *cornu ammonis* (CA) regions of the hippocampus (HPC), the prefrontal cortex (PFC) and the nucleus accumbens (NAc). Moreover, we assessed the expression of these miRNAs in peripheral blood samples of the same animals. Bioinformatics analyses were also performed to get insights into possible gene targets of these miRNAs as well as the pathways and functions in which they are involved.

## Materials and Methods

The array data in which the two herein presented miRNAs were identified are publicly accessible from NCBI/GEO (GSE56028).

### Animals

Male Wistar rats (2-month old; Charles River Laboratories) were maintained under standard laboratory conditions (lights on 08:00–20:00 h; 22°C, relative humidity of 55%, *ad libitum* access to food and water).

#### Unpredictable Chronic Mild Stress (CMS)

Rats (*n* = 10–12/group) were randomly assigned to one of the following groups: non-stressed control (CT) + vehicle (NaCl 0.9%); stress-exposed (CMS) + vehicle; and CMS+fluoxetine (CMS+FLX). A validated CMS protocol was applied for 6 weeks, as previously described (Bessa et al., 2009). During the last 2 weeks of the CMS protocol, animals were injected daily with fluoxetine (intraperitoneal injection; 10 mg/kg in ultra-pure water; Kemprotec, Middlesborough, UK) or vehicle. Fluoxetine dose was chosen based on previous studies (Bessa et al., 2009; Patrício et al., 2015). All procedures were carried out following the EU Directive 2010/63/EU and the Portuguese guidelines on animal care and experimentation.

#### Behavioral Assessments

The development of behavioral signs akin to human depression was assessed using different behavioral tests. Specifically, anxiety-like behavior and anhedonia were evaluated at the end of the CMS protocol, as previously described (Mateus-Pinheiro et al., 2014; Patrício et al., 2015). Anxiety-like behavior was assessed in a novelty suppressed feeding (NSF) paradigm, 72 h before sacrifice and, brain and blood samples collection. Briefly, food-deprived (18 h) animals were placed in an open-field arena with a single food pellet positioned in the center, for a maximum of 10 min. After reaching the pellet, each rat was individually placed in a cage to feed for 10 min. The latency to feed in the open-field arena was used as an anxiety-like behavior index, and the food consumption in the cage provided a measure of appetite drive. Anhedonic behavior was assessed using a modified version of the Sweet Drive test (SDT; Mateus-Pinheiro et al., 2014), 36 h before sacrifice and sample collection. In this test, animals were food-deprived for 10 h following which they were placed in a 3-chamber box and could choose between sugared pellets (Cheerios^®^, Nestlé) or isocaloric regular chow during a 10 min trial. Decreased preference for sugared pellets was taken as a measure of anhedonic behavior.

#### Blood Collection and Corticosterone Levels Measurement

Blood sampling (tail venipuncture) was performed during the diurnal nadir (N, 08:00–09:00) and the diurnal zenith (Z, 20:00 −21:00), at the end of the CMS protocol, on the day before sacrifice. Corticosterone levels were measured in the collected blood serum using a Corticosterone ELISA Kit (ab108821, Abcam), according to the manufacturer’s instructions.

#### Brain Regions Macrodissection

Dorsal (dDG; *n* = 6–9) and ventral DG (vDG; *n* = 6–9), remaining hippocampus (HPC; *n* = 3), prefrontal cortex (PFC; *n* = 3) and nucleus accumbens (NAc; *n* = 3) were collected 24 h after the last stressor/fluoxetine injection. Animals were first anesthetized with pentobarbital and transcardially perfused with 0.9% saline. Immediately after dissection, tissues were frozen and stored at −80°C until further analysis. To avoid experimenter-dependent bias, brains were macrodissected by a single investigator.

#### RNA Purification and Real-Time PCR

Total RNA, including miRNAs, was isolated from the macro-dissected brain regions and blood samples (collected during diurnal nadir, on the day of sacrifice) using the Direct-zol™ RNA MiniPrep (ZymoResearch), according to the manufacturer’s instructions. RNA samples were treated with qScript™ microRNA cDNA Synthesis Kit (Quanta Biosciences) to generate cDNA. QRT-PCR was performed using PerfeCTa microRNA assay for miR-411-5p and miR-409-5p (Quanta Biosciences) and PerfeCTa Universal PCR primer (Quanta Biosciences). Samples were analyzed using 5xHOT FIREPol^®^ EvaGreen^®^ qPCR Mix Plus (ROX, Solis BioDyne), according to the manufacturer’s instructions, in an AB7500 Fast Real-Time PCR system (Applied Biosystems). U6 small nuclear RNA (RNU6) was used as an internal reference (Control Assay, Quanta Biosciences). The results are presented as fold change (2ΔΔCT) of control samples.

#### miRNA Target Prediction and Pathway Analysis

For the computational prediction of miRNAs target genes, the mirWalk web platform database was used^1^ (Sticht et al., 2018). This tool incorporates databases from other established programs for miRNA target prediction. In the present study, the following databases were included for target prediction: mirWalk, miRanda, miRDB, and TargetScan. For functional analysis, we considered the target genes commonly predicted by at least three out of the four databases for each mature miRNA (Accession numbers: miRNA 409-MIMAT0003204 and miRNA 411-MIMAT0005312), using the following parameters for target prediction: Gene region—3′UTR binding site; 2,000 bp upstream flanking region (assumed promoter); minimum seed length: 7 nucleotides; *p* < 0.05). For the functional annotation of miRNA predicted targets, we used the PANTHER database and for interaction analysis, we used the STRING database.

#### Statistical Analysis

Statistical analysis was performed using the GraphPad Prism 8.0 software (GraphPad Software, Inc., La Jolla, CA, USA). The underlying assumptions of all statistical procedures were assessed. The normal distribution was tested using the Kolmogorov–Smirnov test. One-Way ANOVA with Sidak *post hoc* multi comparisons test was used to assess differences between experimental groups in NSF, SDT, and miRNA levels. Two-way ANOVA repeated measures with Sidak *post hoc* multi comparisons test was used to assess differences between groups in corticosterone levels. Test statistics are presented in the text and *post hoc* *p*-values are shown in the Figures. Significance was set at *p* < 0.05.

### Results

**Figure 1:**
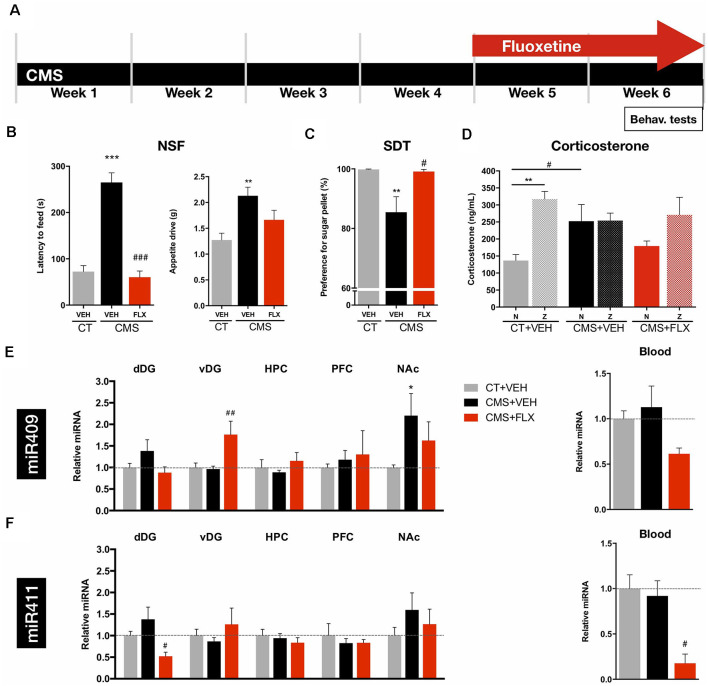
Behavioral analyses and miRNA levels after CMS exposure and fluoxetine treatment. **(A)** CMS protocol was applied for 6 weeks and fluoxetine treatment was performed during the last 2 weeks. Behavioral analyses and blood collection were performed at the end of the CMS protocol. **(B)** Novelty suppressed feeding (NSF) test was applied to evaluate anxiety-like behavior; left panel: latency to feed and right panel: appetite drive. **(C)** Sweet Drive Test (SDT) was used to assess anhedonia. **(D)** Corticosterone levels measured in the blood serum of rats between 8:00 and 9:00 (basal levels, Nadir, N) and between 20:00 and 21:00 (peak levels, Zenith, Z). **(E)** Relative levels of miRNA-409-5p in the dorsal (dDG) and ventral dentate gyrus (vDG), CA regions of the hippocampus (HPC), prefrontal cortex (PFC), nucleus accumbens (NAc), and in the blood. **(F)** Relative levels of miR-411-5p in the dDG, vDG, HPC, PFC, NAc, and in the blood. *Denotes differences between CT and CMS; ^#^Denotes differences between CMS+VEH and CMS+FLX. Abbreviations: CT, Control; CMS, unpredictable chronic mild stress; NSF, Novelty Suppressed Feeding test; SDT, Sweet Drive test. Data presented as mean ± SEM. FLX, fluoxetine; N, Nadir; Z, Zenith. ^*/#^*p* < 0.05; ^**/##^*p* < 0.01; ^***/###^*p* < 0.001.

To assess the levels of the previously identified miRNAs in different brain regions and blood of depressive-like and antidepressant treated animals, we used a validated rat model of depression, the Chronic Mild Stress (CMS; Figure 1A). The behavioral analysis confirmed the development of anxiety-like and anhedonic behavior after CMS exposure, two important components of depressive-like behavior phenotype in this animal model, as shown by the increased latency time to reach the pellet in the NSF test (Figure 1B; *F*_(2,32)_ = 51.29, *p* < 0.001), and by a decreased preference for the sugared pellet in the SDT test (Figure 1C; *F*_(2,32)_ = 6.293, *p* = 0.0052), respectively. On the other hand, chronic treatment, for 2 weeks, with fluoxetine reversed both anxiety-like and anhedonic behavior (Figures 1B,C). In line with these behavioral deficits, and also a relevant hallmark of this animal model, CMS-exposed rats presented significantly higher corticosterone levels in the blood serum during the diurnal Nadir (N; Figure 1D), as compared to control (CT) rats. Fluoxetine treatment was not able to completely reverse the effects of CMS on the diurnal Nadir corticosterone levels back to those of CT animals (N; Figure 1D; Interaction: *F*_(2,33)_ = 3.583, *p* = 0.0391; Timepoint of blood collection: *F*_(1,33)_ = 10.29, *p* = 0.0030).

### miR-409-5p and miR-411-5p Levels Change in the Brain and Blood After Chronic Mild Stress and Fluoxetine Treatment

The levels of miR-409-5p and miR-411-5p were analyzed in brain regions traditionally associated with depression pathophysiology, namely the hippocampus (DG and CA regions), the PFC and the nucleus accumbens (NAc), upon exposure to CMS and after treatment with fluoxetine. Given the functional heterogeneity of the hippocampal formation, we sought to investigate the levels of these miRNA along the DG Spatio-temporal axis. Analysis of miR-409-5p levels in the dorsal (dDG) and ventral DG (vDG) revealed no statistically significant differences between CT and CMS-exposed animals, whereas fluoxetine treatment significantly increased the levels of this miRNA in the vDG, specifically (Figure 1E; dDG: *F*_(2,20)_ = 1.843, *p* = 0.1843; vDG: *F*_(2,20)_ = 7.176, *p* = 0.0045). The levels of miRNA-409-5p were also significantly increased in the NAc of CMS-exposed animals, but not reversed by fluoxetine treatment (Figure 1E; *F*_(2,6)_ = 7.246, *p* = 0.0251). The levels of this miRNA in the CA regions of the HPC, in the PFC (Figure 1E; HPC: *F*_(2,5)_ = 0.5157, *p* = 0.6257; PFC: *F*_(2,6)_ = 0.2143, *p* = 0.8130), or in the blood (Figure 1E, right panel; *F*_(2,6)_ = 3.256, *p* = 0.1103) were not significantly impacted by CMS exposure or fluoxetine treatment.

Regarding miR-411-5p levels, we could not disclose statistically significant differences between CMS-exposed and CT animals in the dDG, though a statistically significant decrease in the levels of this miRNA was observed upon chronic treatment with fluoxetine (Figure 1F; *F*_(2,20)_ = 4.107, *p* = 0.0320). The levels of this miRNA were not impacted by CMS or fluoxetine treatment in any of the remaining brain regions—vDG, CA regions of the HPC, PFC and NAc (Figure 1F; vDG: *F*_(2,20)_ = 0.9404, *p* = 0.4071; HPC: *F*_(2,5)_ = 0.4372, *p* = 0.6684; PFC: *F*_(2,6)_ = 0.3124, *p* = 0.7429; NAc: *F*_(2,6)_ = 2.566, *p* = 0.1566). Strikingly, and in line with the results for the dDG, the levels of this miRNA were significantly decreased in the blood of fluoxetine-treated CMS animals (Figure 1F, right panel;*F*_(2,6)_ = 10.06, *p* = 0.0121).

### Bioinformatics Analysis Reveals Specific Target Genes and Pathways for miR-409-5p and miR-411-5p

**Figure 2:**
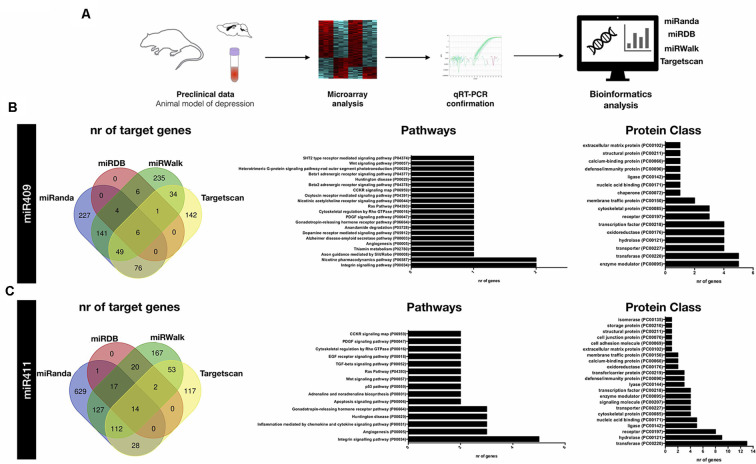
Bioinformatics analyses of miR-409-5p and miR-411-5p target genes. **(A)** Schematic representation of the experimental approach used to identify and analyze miR-409-5p and miR-411-5p target genes. **(B,C)** Venn diagrams depicting the number of target genes identified by each of the miRNA target prediction tools: miRanda, miRDB, miRWalk and TargetScan (left panel), and list of the top significantly enriched pathways and protein classes identified by PANTHER database (right panel) for **(B)** miR-409 and **(C)** miR-411 target genes, respectively. The target genes predicted by three out of the four miRNA target genes predicting tools were used as input in the PANTHER database.

To identify possible target genes for each of these miRNAs, we used 4 different online target prediction tools: miRanda, miRDB, miRWalk, and TargetScan (Figure 2A). Venn diagrams depict the number of predicted genes by each tool (Figures 2B,C). For further bioinformatics analyses, we considered the experimental target genes predicted by at least three out of the four databases. This strategy unveiled 62 and 145 target genes for miR-409-5p and miR-411-5p, respectively (Supplementary Tables S1, S2). Both gene lists were further computed into the PANTHER classification system for functional annotation analysis of the miRNAs target genes. This clustering of the predicted target genes was performed through pathway enrichment and protein class analyses.

Regarding mir-409-5p, we found very few genes assigned to each pathway, with the integrin signaling pathway and nicotine pharmacodynamics pathway being the most represented (two genes in each pathway; Figure 2B). The majority of the predicted target genes for mir-409-5p fell into the enzyme modulator and transferase protein class categories (six genes per category), followed by the transporter, hydrolase, oxidoreductase, and transcription factor protein classes (five genes per category).

**Figure 3:**
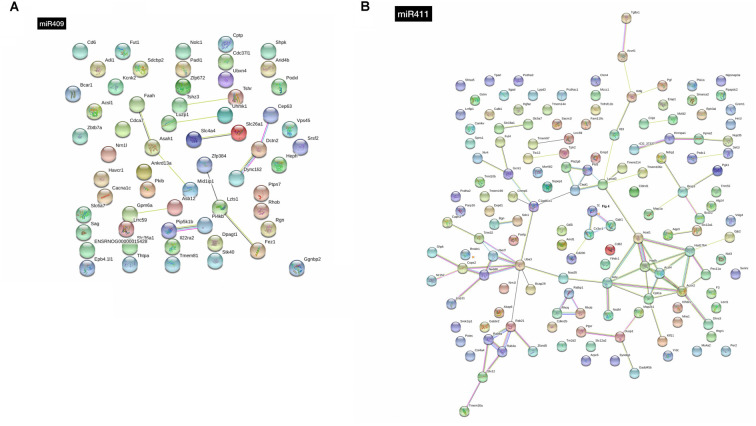
STRING analysis of the interactions between the target genes of **(A)** miR-409-5p and **(B)** miR-411-5p. Thicker lines indicate stronger network connections between the genes.

This target gene prediction analysis for mir-409-5p also identified several genes of the solute carrier family, namely *Slc26a1*, *Slc36a1*, *Slc4a4*, and *Slc6a7* and the Zinc finger protein (ZFP) family, namely *Zfp384*, *Zfp403* and *Zfp672* (Supplementary Table S1). Moreover, some of these genes were represented in the interaction analysis, including *Slc4a4* interacting with *Slc26a1*, and Zfp384, interacting with *Lzts1* and *Fez1* (Figure 3A).

Concerning mir-411-5p target genes, the pathway enrichment analysis identified an over-representation of the integrin signaling, angiogenesis, inflammation mediated by chemokine and cytokine, Huntington disease, and gonadotropin-releasing hormone receptor, related genes (Figure 2C). The most represented protein classes in the protein enrichment analysis for the mir-411-5p target genes were transferase (13 genes), hydrolase (nine genes), and receptor (eight genes; Figure 2C). In the list of 145 mir-411-5p target genes identified in three out of four target prediction databases, several solute carrier family genes, including *Slc12a1*, S*cl12a2*, *Slc18a1*, and *Slc5a7* was also detected, similar to what was found for mir-409-5p. Also, three genes of the transmembrane protein family were represented, *Tmem106c*, *Tmem30a*, and *Tmem97* (Supplementary Table S2). The STRING analysis revealed a few clusters of interacting genes, including: (i) *Rab9a*, *Rab21, Rab4a, Stx12* and *Tmem30a*; (ii) *Dusp1, Ptprr, Map2k1* and *Gadd45b*, (iii) *Acsl4, Acat1, Cpt1a, Acox2, Hsd17b*4 and Pex11a (Figure 3B).

## Discussion

In a previous genome-wide analysis, we identified two pre-miRNAs—miR-409 and miR-411—to be significantly upregulated in the whole hippocampal DG of CMS-exposed rats when compared to the Control rats. Moreover, their levels were restored by chronic treatment with antidepressants from four different classes (fluoxetine, imipramine, tianeptine, and agomelatine; Patrício et al., 2015). Here, we investigated the levels of miR-409-5p and miR-411-5p along the septotemporal axis of the DG and in other brain regions associated with depression pathophysiology, including the CA regions of the hippocampus, the PFC and the NAc, and in peripheral blood samples. To further explore the relevance of these miRNAs for molecular regulation and brain function, we performed bioinformatics analysis to identify possible gene targets and functional analysis of pathways and protein functions.

First, we observed that the impact of CMS exposure on the levels of both miRNAs in each of the brain regions analyzed was very modest, with a similar pattern observed for both miRNAs and with only statistically significant increases in mir-409-5p levels in the NAc. Whereas our previous microarray data had shown increased levels of both miRNAs in the DG upon CMS exposure, here we could only detect a moderate trend for this increase in the dDG for both miRNAs, suggesting this subregion to be the major contributor for the effect previously observed in the whole DG samples (Patrício et al., 2015). Chronic fluoxetine treatment effects were more pronounced than those of the CMS alone, and its impact on the levels of the two miRNAs among brain regions was very heterogeneous. We show that fluoxetine treatment significantly increased the levels of mir-409-5p in the vDG while inducing major decreases in the levels of mir-411-5p in both the dDG and in the blood. This contrasting impact of fluoxetine on the two DG poles was not unexpected, in light of the functional and molecular dichotomy along the hippocampal longitudinal axis (Strange et al., 2014) and may reflect also functional differences between both miRNAs. Moreover, and though the main goal of this work was to understand the changes induced by antidepressants in a pathological context, by modeling the human depressive condition, these fluoxetine effects encourage the analysis of mir-411-5p levels in the brain of naïve animals (not exposed to stress) treated with fluoxetine.

The potential use of miRNAs as biomarkers of disease and treatment response (Dalton et al., 2014), in part due to their stability in bodily fluids that can be collected by minimally invasive procedures, is encouraged by gene expression studies showing similarities between blood and brain samples (Liew et al., 2006; Chen et al., 2008; Jasinska et al., 2009). Here, we show similarities between fluoxetine impact on the dDG and peripheral blood, though the origin and functional role of these miRNAs in the blood are yet to be fully determined (Scott et al., 2015).

Previous studies have shown how miRNAs can regulate antidepressant treatment, namely selective serotonin reuptake inhibitors (SSRIs), as is the case of fluoxetine, the antidepressant used in this study (Baudry et al., 2010; Issler et al., 2014). At least two other miRNAs, mir-16 and mir-135, were shown to be involved in the action of SSRIs in depressed individuals, by targeting genes related to brain serotoninergic system, including *Slc6a4* [serotonin (5-HT) transporter, responsible for 5-HT reuptake (SERT) and *Htr1a* (5-HT inhibitory receptor 1a; Issler et al., 2014)]. Interestingly enough, *Slc18a1*, or vesicular monoamine transporter 1 (VMAT1), which is involved in the packaging and storage of serotonin in presynaptic terminals, is among the mir-411-5p predicted target genes, identified in our *in silico* analysis. Moreover, *Slc18a1* has been implicated in the development and treatment of psychiatric disorders (Lohoff et al., 2006; Lin et al., 2011), further suggesting a functional role of mir-411-5p in this context.

Functional annotation analysis of miR-409-5p target genes also revealed, a group of Zinc-finger protein (ZFP) genes. The ZFP family is a large group of proteins, capable of binding nucleic acids, proteins, or small molecules, involved in the regulation of many cellular processes (Cassandri et al., 2017). Many of these genes have been associated with neuropsychiatric disorders including schizophrenia, bipolar disease, and intellectual disability (Sun et al., 2015). In particular, the expression of one of these target genes—*Zfp672-* has been previously reported to be increased in the brains of Low Anxiety Behavior (LAB) mice, an animal model of anxiety-trait behavior, compared to High Anxiety Behavior mice (Czibere et al., 2011). In the CMS model of depression, we also observe the concomitant emergence of anxiety-like behavior, which is reversed by fluoxetine treatment. This behavioral outcome and the increased levels of miR-409-5p in the vDG, a brain region that has been implicated in anxiety behavior, upon fluoxetine treatment, reinforce an involvement of *Zpf672* in the development or treatment of this trait. Another ZFP identified here was *Zfp384*, which has been shown to contribute to the regulation of the dendritic growth of newborn hippocampal neurons (Kang et al., 2011). Our previous studies with this animal model have consistently shown the impact of both chronic stress and fluoxetine treatment in dendritic morphology (Bessa et al., 2009, 2013; Mateus-Pinheiro et al., 2013b; Patrício et al., 2015). This is true, namely in the brain regions where changes in the levels of this miRNA were observed, the vDG and the NAc. In line with the possible role of these miRNAs in regulating neural plasticity, one of the identified target genes for mir-411-5p was Dual specificity protein phosphatase 1 (*Dusp1*), a negative regulator of the MAP kinase pathway (Huang and Tan, 2012). This gene was found to be upregulated upon CMS exposure and reversed by antidepressant treatment, in our previous microarray analysis (Patrício et al., 2015), and has been associated with depression pathophysiology in human patients and in an animal model of depression (Duric et al., 2010). String analysis showed that *Dusp1, Gadd45b*, and *Map2k1*, three predicted target genes of miR-411-5p, clustered together in terms of their possible interactions. Gadd45b is a DNA demethylating agent that regulates expression of *Bdnf* and *Fgf1* and has been shown to influence synaptic plasticity and memory processes, while also mediating the effects of social stress in the mesolimbic dopamine circuit (Ma et al., 2009; Mateus-Pinheiro et al., 2011; Labonté et al., 2019). *Map2k1 (Mek1)* encodes a kinase of the MAPK/ERK pathway, that has been implicated in synaptic plasticity processes and memory, through activation of MAPK signaling (Kelleher et al., 2004). A recent study also reported MAP2K1 to regulate Neuronal Per Arnt Sim domain protein 4 (NPAS4), an immediate-early gene that controls a transcriptional program involving neural activity-regulated genes (Lin et al., 2008; Yun et al., 2013), including *Bdnf*, thus promoting neural circuitry plasticity, learning, and memory (Funahashi et al., 2019). Interestingly, our previous work revealed that *Npas4* is epigenetically regulated in a conditional *Tet3* KO mouse model that presents anxiety-like behavior and cognitive deficits (Antunes et al., 2020). Changes in epigenetic regulators have long been described in the context of stress, depression and antidepressant treatment (Nestler, 2014), with chronic stress being suggested to interact with susceptibility genes *via* epigenetic mechanisms to produce long-lasting changes in the brain that may partly explain the heterogeneity of depression etiology (Tsankova et al., 2007; Mateus-Pinheiro et al., 2011; Menke and Binder, 2014). miRNAs, in particular, have emerged as an important form of epigenetic regulation of gene expression in the context of depression, with growing evidence suggesting their role in both the pathophysiology and treatment of this disorder (Hansen and Obrietan, 2013; Issler et al., 2014; Maffioletti et al., 2014).

Here, we do not provide enough experimental evidence to support the causal relationship between these changes in the levels of miRNAs and the neural plasticity changes that have been previously shown to underlie some of the behavioral deficits observed in this animal model, including neuronal morphology changes and decreased hippocampal cytogenesis (Bessa et al., 2009, 2013; Mateus-Pinheiro et al., 2013a). In this work, we have not analyzed the expression of putative target genes identified in the *in silico* analysis. Though this is a valid and common approach to address the functional role of miRNAs, it might also raise some questions and limit the interpretation of the data, because there may not be a direct association between the levels of miRNAs and of their target genes. First, miRNAs may act either as enhancers or repressors of gene expression by binding to distinct regulatory regions of the genome (O’Brien et al., 2018). In fact, under certain circumstances and if interacting at the promoter level, miRNAs can also activate transcription (Dharap et al., 2013). Second, because it is not straightforward to negatively or positively associate the levels of a regulatory molecule *in vivo* with the levels of its targets or effectors as one might not exclude the possibility of compensatory mechanisms. High target gene levels may either trigger an increase in the cognate miRNA levels or be the reflection of that miRNA low levels. Nevertheless, by combining miRNAs levels assessment with bioinformatics analysis of the predicted targets, we hypothesize that these miRNAs, may be important mediators of the effects of chronic stress and fluoxetine, in neural plasticity. Functional assays, using pre-miRs and anti-miRs, would help disclose any causal effect. We additionally highlight the need to further explore the levels of the mature miR-3p form of both miRNAs in this context.

Despite the animal model used herein is based in a very broad stimulus known to induce depressive-like signs in rodents—chronic stress—and the fact that the levels of these two miRNAs had been previously shown to be impacted by different classes of antidepressants, may reflect that these effects may be generalized to other stress-based animal models and antidepressants. Thus, these findings need further validation in other animal models, including in female subjects, and also in clinical samples. Still, they encourage further investigation of miRNAs as targets for disruption and treatment prediction in the context of depression, towards a better understanding of the neurobiology of the disease and more precise diagnosis and directed treatment.

## Data Availability Statement

Publicly available datasets were analyzed in this study. This data can be found here: https://www.ncbi.nlm.nih.gov/geo/query/acc.cgi?acc=GSE56028.

## Ethics Statement

The animal study was reviewed and approved by ORBEA EM/ICVS-I3Bs School of Medicine, University of Minho, Braga, Portugal.

## Author Contributions

PP and LP designed the study. PP and AM-P performed all the experiments. NA and MM assisted in the CMS protocol, sacrifice, brain, and blood samples collection and processing. PP wrote the manuscript. JB, AR, NS, and LP revised the manuscript.

## Conflict of Interest

The authors declare that the research was conducted in the absence of any commercial or financial relationships that could be construed as a potential conflict of interest.

## Footnotes

^1^http://www.umm.uni-heidelberg.de/apps/zmf/mirwalk/
