# Supplementary material for: miR-409 and miR-411 Modulation in the Adult Brain of a Rat Model of Depression and After Fluoxetine Treatment
Source: Front Behav Neurosci. 2020 Aug 7;14:136. doi: 10.3389/fnbeh.2020.00136 (PMC7427047; doi:10.3389/fnbeh.2020.00136)
Supplement: Supplementary file 1 [file Table_1.docx]

Supplementary Material

**Supplementary** **table 1:** List of the commonly predicted target genes for miRNA-409-5p, using 4 databases (miRanda, miRDB, miRWalk and TargetScan). Number 1 indicates that gene was identified in the database.

|  | **Target prediction tool** | | | |
| --- | --- | --- | --- | --- |
| **Gene** | **miRanda** | **miRDB** | **miRWalk** | **Targetscan** |
| *Acsl1* | 1 | 0 | 1 | 1 |
| *Adi1* | 1 | 0 | 1 | 1 |
| *Ankrd13* | 1 | 0 | 1 | 1 |
| *Arid4b* | 1 | 1 | 1 | 1 |
| *Asah1* | 1 | 0 | 1 | 1 |
| *Asb12* | 1 | 0 | 1 | 1 |
| *Bcar1* | 1 | 0 | 1 | 1 |
| *Cacna1c* | 1 | 0 | 1 | 1 |
| *Carkl* | 1 | 0 | 1 | 1 |
| *Cd6* | 1 | 0 | 1 | 1 |
| *Cdc37l1* | 1 | 1 | 1 | 1 |
| *Cdca7* | 1 | 0 | 1 | 1 |
| *Dctn2* | 1 | 0 | 1 | 1 |
| *Dncli2* | 1 | 0 | 1 | 1 |
| *Dpagt1* | 1 | 0 | 1 | 1 |
| *Epb4.1l1* | 1 | 0 | 1 | 1 |
| *Faah* | 1 | 1 | 1 | 0 |
| *Fez1* | 1 | 0 | 1 | 1 |
| *Fut1* | 1 | 0 | 1 | 1 |
| *Gpm6a* | 1 | 1 | 1 | 1 |
| *Havcr1* | 1 | 0 | 1 | 1 |
| *Heph* | 1 | 1 | 1 | 0 |
| *Il22ra2* | 1 | 0 | 1 | 1 |
| *Kcnk2* | 1 | 0 | 1 | 1 |
| *LOC300963* | 1 | 0 | 1 | 1 |
| *LOC361399* | 1 | 0 | 1 | 1 |
| *LOC498229* | 1 | 1 | 1 | 0 |
| *Lrrc59* | 1 | 0 | 1 | 1 |
| *Luzp1* | 1 | 0 | 1 | 1 |
| *Lyk4* | 1 | 0 | 1 | 1 |
| *Lzts1* | 1 | 0 | 1 | 1 |
| *MGC94339* | 1 | 0 | 1 | 1 |
| *Mig12* | 1 | 0 | 1 | 1 |
| *Nolc1* | 1 | 0 | 1 | 1 |
| *NP_001100976.1* | 0 | 1 | 1 | 1 |
| *Padi1* | 1 | 0 | 1 | 1 |
| *Pip5k1b* | 1 | 0 | 1 | 1 |
| *Pik4cb* | 1 | 0 | 1 | 1 |
| *Pkib* | 0 | 1 | 1 | 1 |
| *Pkib* | 1 | 0 | 1 | 1 |
| *Podxl* | 1 | 0 | 1 | 1 |
| *Ptpn7* | 1 | 0 | 1 | 1 |
| *RGD1310230* | 1 | 0 | 1 | 1 |
| *Rgn* | 1 | 1 | 1 | 1 |
| *Rhob* | 1 | 0 | 1 | 1 |
| *Sag* | 1 | 0 | 1 | 1 |
| *Sdcbp2* | 1 | 0 | 1 | 1 |
| *Sfrs2* | 1 | 1 | 1 | 1 |
| *Slc26a1* | 1 | 0 | 1 | 1 |
| *Slc36a1* | 1 | 0 | 1 | 1 |
| *Slc4a4* | 1 | 0 | 1 | 1 |
| *Slc6a7* | 1 | 0 | 1 | 1 |
| *Thtpa* | 1 | 0 | 1 | 1 |
| *Tshr* | 1 | 1 | 1 | 0 |
| *Ubxd2* | 1 | 0 | 1 | 1 |
| *Uhmk1* | 1 | 0 | 1 | 1 |
| *Vps45* | 1 | 0 | 1 | 1 |
| *Zbtb7a* | 1 | 0 | 1 | 1 |
| *Zfp384* | 1 | 0 | 1 | 1 |
| *Zfp384* | 1 | 0 | 1 | 1 |
| *Zfp403* | 1 | 0 | 1 | 1 |
| *Zfp672* | 1 | 0 | 1 | 1 |

**Supplementary** **table 2:** List of the commonly predicted target genes for miRNA-411-5p, using 4 databases (miRanda, miRDB, miRWalk and TargetScan). Number 1 indicates that gene was identified in the database.

|  | **Target prediction tool** | | | |
| --- | --- | --- | --- | --- |
| **Gene symbol** | **miRanda** | **miRDB** | **miRWalk** | **Targetscan** |
| *Acat1* | 1 | 0 | 1 | 1 |
| *Acly* | 1 | 0 | 1 | 1 |
| *Acox2* | 1 | 1 | 1 | 0 |
| *Acsl4* | 1 | 0 | 1 | 1 |
| *Acvrl1* | 1 | 0 | 1 | 1 |
| *Akap6* | 1 | 0 | 1 | 1 |
| *Amd1* | 1 | 0 | 1 | 1 |
| *Aqp3* | 1 | 0 | 1 | 1 |
| *Arpc5* | 1 | 0 | 1 | 1 |
| *Arts1* | 1 | 1 | 1 | 0 |
| *Bcap29* | 1 | 0 | 1 | 1 |
| *Bcl2l2* | 1 | 0 | 1 | 1 |
| *Bgn* | 1 | 0 | 1 | 1 |
| *Bnip3* | 1 | 1 | 1 | 1 |
| *C1galt1c1* | 1 | 0 | 1 | 1 |
| *Calb1* | 1 | 0 | 1 | 1 |
| *Camkv* | 1 | 0 | 1 | 1 |
| *Capn3* | 1 | 0 | 1 | 1 |
| *Carkl* | 1 | 0 | 1 | 1 |
| *Cd200* | 1 | 0 | 1 | 1 |
| *Cd82* | 1 | 0 | 1 | 1 |
| *Cdkn2b* | 1 | 0 | 1 | 1 |
| *Cept1* | 1 | 0 | 1 | 1 |
| *Chmp5* | 1 | 0 | 1 | 1 |
| *Clcn4-2* | 1 | 0 | 1 | 1 |
| *Cldnd1* | 1 | 0 | 1 | 1 |
| *Col4a4* | 1 | 0 | 1 | 1 |
| *Cops2* | 1 | 0 | 1 | 1 |
| *Cpt1a* | 1 | 1 | 1 | 0 |
| *Cript* | 1 | 1 | 1 | 0 |
| *Cx3cr1* | 1 | 0 | 1 | 1 |
| *Dhrs3* | 1 | 0 | 1 | 1 |
| *Dusp1* | 1 | 1 | 1 | 1 |
| *Edg1* | 1 | 0 | 1 | 1 |
| *Emp2* | 1 | 0 | 1 | 1 |
| *F3* | 1 | 0 | 1 | 1 |
| *Faslg* | 1 | 0 | 1 | 1 |
| *Fut4* | 1 | 0 | 1 | 1 |
| *Gabbr2* | 1 | 0 | 1 | 1 |
| *Gadd45b* | 1 | 0 | 1 | 1 |
| *Galr1* | 1 | 0 | 1 | 1 |
| *Gclm* | 1 | 1 | 1 | 1 |
| *Gcnt1* | 1 | 0 | 1 | 1 |
| *Gjb2* | 1 | 0 | 1 | 1 |
| *Grem1* | 1 | 0 | 1 | 1 |
| *Gtf2i* | 1 | 0 | 1 | 1 |
| *Hadhsc* | 1 | 0 | 1 | 1 |
| *Hgfac* | 1 | 0 | 1 | 1 |
| *Hnrpa1* | 1 | 0 | 1 | 1 |
| *Hsd17b4* | 1 | 0 | 1 | 1 |
| *Ifngr1* | 1 | 0 | 1 | 1 |
| *Isrip* | 1 | 0 | 1 | 1 |
| *Itgad* | 1 | 0 | 1 | 1 |
| *Kitl* | 1 | 0 | 1 | 1 |
| *Klhdc2* | 1 | 0 | 1 | 1 |
| *Kpna2* | 1 | 0 | 1 | 1 |
| *Lect1* | 1 | 1 | 1 | 1 |
| *LOC303332* | 1 | 0 | 1 | 1 |
| *LOC312102* | 1 | 0 | 1 | 1 |
| *LOC361399* | 1 | 0 | 1 | 1 |
| *LOC500419* | 1 | 0 | 1 | 1 |
| *LOC500638* | 1 | 1 | 1 | 0 |
| *Lrrc59* | 1 | 0 | 1 | 1 |
| *Lrrfip1* | 1 | 1 | 1 | 0 |
| *Lypd3* | 1 | 0 | 1 | 1 |
| *Mak10* | 1 | 0 | 1 | 1 |
| *Map2k1* | 1 | 1 | 1 | 1 |
| *Mboat5* | 1 | 0 | 1 | 1 |
| *Mccc1* | 1 | 0 | 1 | 1 |
| *Mcfd2* | 1 | 0 | 1 | 1 |
| *MGC94600* | 1 | 0 | 1 | 1 |
| *Mns1* | 1 | 0 | 1 | 1 |
| *Morf4l2* | 1 | 0 | 1 | 1 |
| *Ms4a2* | 1 | 1 | 1 | 1 |
| *Mtap1a* | 1 | 0 | 1 | 1 |
| *Ndrg1* | 1 | 1 | 1 | 1 |
| *Nedd8* | 1 | 0 | 1 | 1 |
| *Nipsnap3a* | 1 | 0 | 1 | 1 |
| *Nol3* | 1 | 0 | 1 | 1 |
| *NP_001099826.1* | 0 | 1 | 1 | 1 |
| *NP_001102021.1* | 0 | 1 | 1 | 1 |
| *Nr1h2* | 1 | 0 | 1 | 1 |
| *Nsdhl* | 1 | 0 | 1 | 1 |
| *Nup35* | 1 | 0 | 1 | 1 |
| *Parp16* | 1 | 0 | 1 | 1 |
| *Pcdha2* | 1 | 1 | 1 | 1 |
| *Pcdha2_predicted* | 1 | 1 | 1 | 1 |
| *Pcdha3* | 1 | 1 | 1 | 0 |
| *Pcdhac2* | 1 | 1 | 1 | 1 |
| *Per2* | 1 | 0 | 1 | 1 |
| *Pex11a* | 1 | 1 | 1 | 1 |
| *Pgf* | 1 | 0 | 1 | 1 |
| *Pgk1* | 1 | 0 | 1 | 1 |
| *Pla2g6* | 1 | 0 | 1 | 1 |
| *Pld3* | 1 | 0 | 1 | 1 |
| *Ppapdc2* | 1 | 1 | 1 | 0 |
| *Pspla1* | 1 | 0 | 1 | 1 |
| *Ptprr* | 1 | 0 | 1 | 1 |
| *Rab21* | 1 | 1 | 1 | 1 |
| *Rab4a* | 1 | 0 | 1 | 1 |
| *Rab9* | 1 | 0 | 1 | 1 |
| *Ralbp1* | 1 | 0 | 1 | 1 |
| *Rg9mtd3* | 1 | 1 | 1 | 0 |
| *RGD1305117* | 1 | 0 | 1 | 1 |
| *RGD1305356* | 1 | 0 | 1 | 1 |
| *RGD1305613* | 1 | 0 | 1 | 1 |
| *RGD1307401* | 1 | 0 | 1 | 1 |
| *RGD1308454* | 1 | 1 | 1 | 0 |
| *RGD1310753* | 1 | 1 | 1 | 0 |
| *RGD1311155* | 1 | 0 | 1 | 1 |
| *RGD1311307* | 1 | 1 | 1 | 0 |
| *RGD1311324* | 1 | 0 | 1 | 1 |
| *RGD1312003* | 1 | 0 | 1 | 1 |
| *Rhob* | 1 | 0 | 1 | 1 |
| *Rhoq* | 1 | 0 | 1 | 1 |
| *Rnf29* | 1 | 0 | 1 | 1 |
| *Rnpc2* | 1 | 1 | 1 | 1 |
| *Rph3al* | 1 | 0 | 1 | 1 |
| *Sacm1l* | 1 | 0 | 1 | 1 |
| *Scpep1* | 1 | 0 | 1 | 1 |
| *Sdc1* | 1 | 0 | 1 | 1 |
| *Sel1h* | 1 | 0 | 1 | 1 |
| *Slc12a1* | 1 | 0 | 1 | 1 |
| *Slc12a2* | 1 | 0 | 1 | 1 |
| *Slc18a1* | 1 | 0 | 1 | 1 |
| *Slc5a7* | 1 | 0 | 1 | 1 |
| *Smarca2* | 1 | 1 | 1 | 0 |
| *Stx12* | 1 | 1 | 1 | 0 |
| *Tcfcp2l2* | 1 | 1 | 1 | 1 |
| *Tgfbr1* | 1 | 0 | 1 | 1 |
| *Tm2d2* | 1 | 0 | 1 | 1 |
| *Tmem106c* | 1 | 0 | 1 | 1 |
| *Tmem30a* | 1 | 0 | 1 | 1 |
| *Tmem97* | 1 | 0 | 1 | 1 |
| *Tnfrsf11b* | 1 | 0 | 1 | 1 |
| *Tparl* | 1 | 0 | 1 | 1 |
| *Tph2* | 1 | 1 | 1 | 0 |
| *Tpo1* | 1 | 1 | 1 | 0 |
| *Trim32* | 1 | 0 | 1 | 1 |
| *Ttc12* | 1 | 0 | 1 | 1 |
| *Ube1c* | 1 | 1 | 1 | 0 |
| *Ube2f* | 1 | 0 | 1 | 1 |
| *Usp11* | 1 | 0 | 1 | 1 |
| *XRG4* | 1 | 0 | 1 | 1 |
| *Yt521* | 1 | 0 | 1 | 1 |
